# A method for small-area estimation of population mortality in settings affected by crises

**DOI:** 10.1186/s12963-022-00283-6

**Published:** 2022-01-11

**Authors:** Francesco Checchi, Adrienne Testa, Amy Gimma, Emilie Koum-Besson, Abdihamid Warsame

**Affiliations:** Department of Infectious Disease Epidemiology, Faculty of Epidemiology and Population Health, London School of Hygiene and Tropical Medicine, London, UK

**Keywords:** Mortality, Death rate, Crisis, Humanitarian, Displaced, Emergency, War, Predictive model, Small area estimation, Secondary data, Method

## Abstract

**Background:**

Populations affected by crises (armed conflict, food insecurity, natural disasters) are poorly covered by demographic surveillance. As such, crisis-wide estimation of population mortality is extremely challenging, resulting in a lack of evidence to inform humanitarian response and conflict resolution.

**Methods:**

We describe here a ‘small-area estimation’ method to circumvent these data gaps and quantify both total and excess (i.e. crisis-attributable) death rates and tolls, both overall and for granular geographic (e.g. district) and time (e.g. month) strata. The method is based on analysis of data previously collected by national and humanitarian actors, including ground survey observations of mortality, displacement-adjusted population denominators and datasets of variables that may predict the death rate. We describe the six sequential steps required for the method’s implementation and illustrate its recent application in Somalia, South Sudan and northeast Nigeria, based on a generic set of analysis scripts.

**Results:**

Descriptive analysis of ground survey data reveals informative patterns, e.g. concerning the contribution of injuries to overall mortality, or household net migration. Despite some data sparsity, for each crisis that we have applied the method to thus far, available predictor data allow the specification of reasonably predictive mixed effects models of crude and under 5 years death rate, validated using cross-validation. Assumptions about values of the predictors in the absence of a crisis provide counterfactual and excess mortality estimates.

**Conclusions:**

The method enables retrospective estimation of crisis-attributable mortality with considerable geographic and period stratification, and can therefore contribute to better understanding and historical memorialisation of the public health effects of crises. We discuss key limitations and areas for further development.

**Supplementary Information:**

The online version contains supplementary material available at 10.1186/s12963-022-00283-6.

## Background

### Mortality estimation in crisis-affected populations

In populations exposed to conditions of crisis (armed conflict, food insecurity, natural disasters, etc.), estimates of population mortality provide a basis on which to predicate an appropriate humanitarian response [1, 2], and support advocacy and historical documentation [3, 4]. Over the past two decades, estimates of mortality have informed war crime prosecution in the former Yugoslavia [5], illuminated the toll of armed conflict in Darfur [6, 7], the Democratic Republic of Congo [8] and Iraq [9, 10], documented the impact of famine in Somalia [11] and, most recently, demonstrated the direct and indirect health impacts of the SARS-CoV-2 pandemic [12–14].

Crisis-attributable mortality is difficult to estimate, even in high-income countries [15, 16]. In low-income and/or insecure settings, additional challenges [4, 17] arise, including (i) lack of robust vital events registration; (ii) unfeasibility of representative primary data collection due to insecurity, lack of authorisations, funding constraints or other factors; and (iii) inability to collect robust retrospective data due to having to elicit information on demographic events over a long period in the past (e.g. > 2 years). Response bias as questionnaires probe farther back in time, plus survival and selection biases caused by households disintegrating due to high mortality or migration, challenge survey validity [17]. Establishing a counterfactual (i.e. non-crisis) death rate presents a further challenge, particularly in very protracted crises (e.g. Afghanistan or the eastern Democratic Republic of Congo) where such a baseline has been unobservable for decades.

### Scope of this paper

Here, we describe the design and implementation of a method that addresses the above challenges, and estimates crisis-attributable death rates and tolls based on previously collected data. Applications of previous iterations of the method in Somalia (2010–2012) [11] and South Sudan (2013–2018) [18] have been published elsewhere. Further applications in Somalia (2014–2018), Nigeria and the Democratic Republic of the Congo will be published separately. South Sudan, Somalia and Nigeria examples are however used here to illustrate the application and constraints of the method.

## General design

### Why a small-area estimation approach?

Small-area estimation was developed in the United States to estimate characteristics of interest, e.g. smoking prevalence or poverty levels, for small geographical units (e.g. counties) without having to conduct primary data collection within each such unit [19]. Our method is designed to deliver estimates for small geographical and time strata based solely on existing data.

### General framework

Crisis-attributable mortality can be defined conceptually as the difference between the number (or rate) of deaths that has actually occurred during the crisis and the number (rate) that would have occurred in the absence of the crisis.

**Fig. 1:**
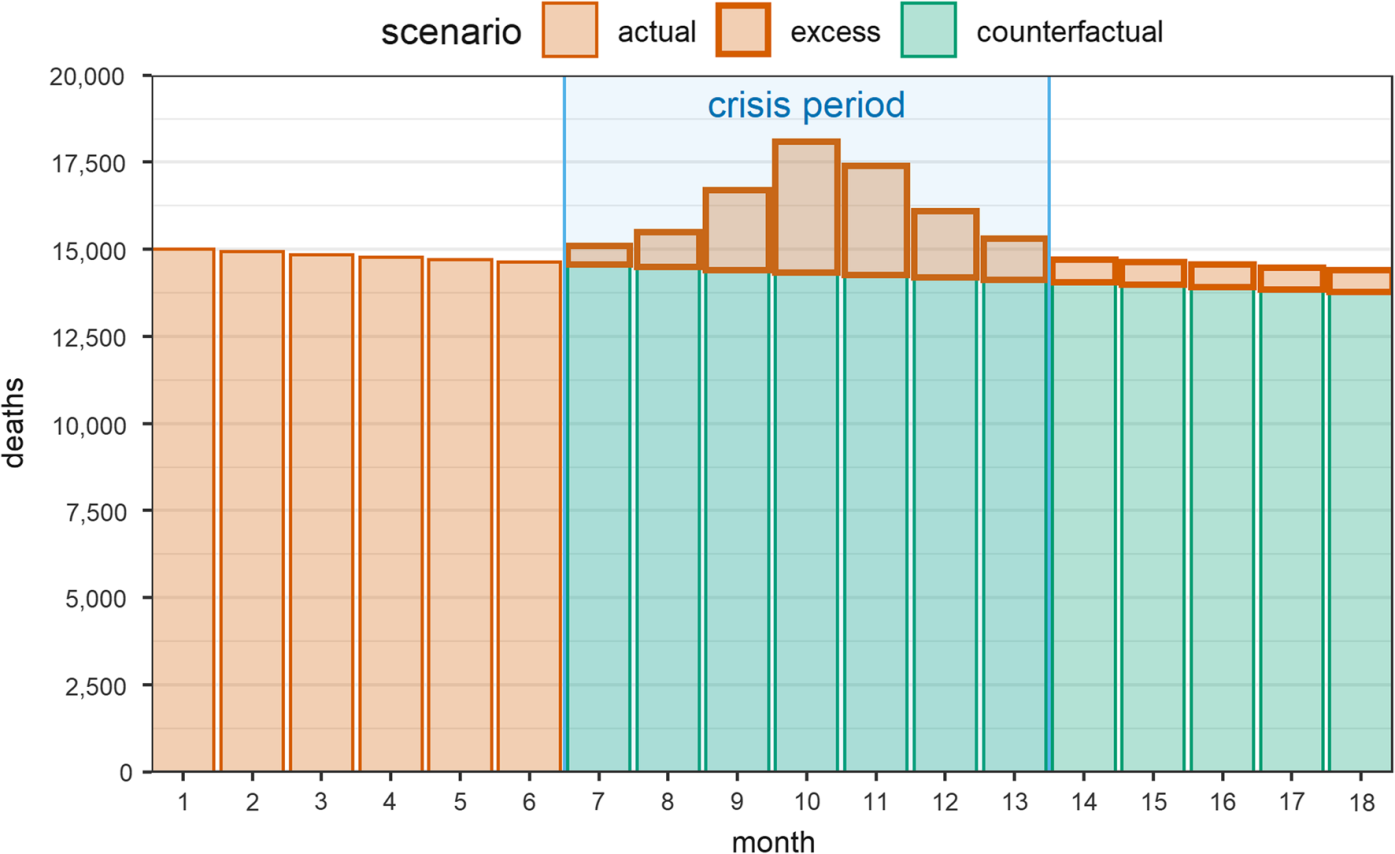
Illustration of actual and counterfactual mortality during and after a hypothetical crisis

As illustrated hypothetically in Fig. 1, in a counterfactual (i.e. no-crisis) scenario it is plausible that the pre-crisis secular decline would have continued; the crisis has negated these improvements and effectively returned the population to a ‘higher’ baseline than pre-crisis; moreover, excess, crisis-attributable mortality may occur even years after crisis conditions (e.g. armed conflict) resolve (e.g. increased tuberculosis mortality due to higher transmission of *M. tuberculosis* when people lived in displacement camps years earlier, or the multi-generational effects of psychological stress).

We wish to estimate excess mortality for the entire ‘person-time’ at risk during the crisis, but also for specific sub-periods and geographic units (these could be administrative level 2 entities such as counties or districts; they could also however be geographical units whose boundaries may correlate more closely with mortality risk, such as settlements for internally displaced persons (IDPs) or ‘livelihood zones’, namely areas characterised by a dominant economic activity, e.g. pastoralism or agriculture). Information on where and when mortality is highest may be useful to identify gaps in the humanitarian response or to better understand the dynamics of an armed conflict. More generally, we can write

1 $$D_{E,kt} = D_{A,kt} - D_{C,kt} = y_{A,kt} N_{A,kt} - y_{C,kt} N_{C,kt}$$

where $D$ is the death toll, $y$ is the mean death rate and $N$ the population at risk; $E$, $A$ and $C$ denote excess, actual (i.e. what truly happened) and counterfactual (what would have happened in the absence of a crisis) levels; $k$ is any geographic unit (e.g. a district), and $t$ any time unit (e.g. a month) within the crisis period (thus, $kt$, the smallest analysis stratum, could be a district-month). Note that ${N}_{C}$ may differ from ${N}_{A}$, for example because in a no-crisis counterfactual forced displacement would not have occurred. If the quantities on the right-hand side of Eq. (1) are all estimated, we can sum results for any $kt$ strata for different aggregations of interest or to compute the overall death toll. Equation (1) also applies for age- or cause-specific mortality (e.g. among children under 5 years old; due to intentional injury), provided these stratifications are available or can also be estimated.

### Estimation steps

Our adaptation of small-area estimation consists of using available data to fit and validate a statistical model (specific to each crisis) that predicts the death rate ${y}_{kt}$ as a function of several predictor variables; and applying this model to project ${y}_{A,kt}$ and ${y}_{C,kt}$ under actual (observed) and assumed counterfactual conditions. Separately, ${N}_{A,kt}$ and ${N}_{C,kt}$ are reconstructed based on growth rates and displacement patterns. Excess deaths are then computed by applying Eq. (1).

Table 1 summarises the steps involved in the full application of the method. Data management details are omitted here, but annotated on R statistical scripts (see Declarations and Additional file 1: pages 11–13). Step 2, namely reconstructing population denominators, will be detailed in a separate paper.

**Table 1:** Summary of estimation steps

Step	Description	Sub-steps	Data requirements	Depends on
*Data collection and management steps*				
1	Identify existing ground mortality data and prepare them for analysis	Identify all available estimates Extract meta-data for each estimate Clean and re-analyse datasets Grade estimate quality Describe data coverage and crude patterns in key demographic indicators	Raw datasets of surveys or other estimation exercises Survey reports Official administrative data, shape files for geographic boundaries	
2	Reconstruct population denominators [not presented in this paper]	Identify and curate alternative population datasets. Grade their robustness Identify and curate displacement data Make appropriate assumptions on population and displacement dynamics Reconstruct population for each $kt$ stratum as an average of alternative estimates	Population datasets Remote sensing estimates Internal and refugee displacement data Explanatory accompanying documents and reports	
3	Capture predictor variable data and prepare them for analysis	Identify possible sources of data based on a conceptual framework Capture and curate predictor datasets Ascertain missingness and perform any appropriate imputation Convert absolute figures into population rates, smooth time series and create lags if appropriate	Predictor datasets Explanation of variable meanings/variable dictionaries	Steps 1–2
*Analysis steps*				
4	Fit a statistical model to predict the death rate as a function of the predictors	Explore correlation among predictors Do univariate analysis Fit alternative multivariate models and select the most appropriate one		Steps 1–3
5	Apply the model to estimate excess mortality while propagating known sources of error	Specify a set of counterfactual scenarios: Agree on what key deviations from normal define the crisis being analysed Arbitrarily define alternative (e.g. most likely, best-case, worst-case) scenarios for what values the model predictors would have taken in the absence of a crisis Construct counterfactual predictor datasets accordingly Apply counterfactual death rates and assumptions on displacement to reconstruct corresponding counterfactual population denominators Set up statistical simulation that implements Eq. (1) for each $kt$ stratum while drawing from known error distributions of each parameter Compute excess death toll estimates overall and for sub-populations/periods of interest	Extensive contextual knowledge Mortality and predictor data for periods as long as possible before the crisis (recommended)	Steps 2–4
6	Conduct sensitivity analyses of interest	Explore how possible bias or uncertainty in key parameters affect the estimates, by running the analysis with alternative data or assumptions		Step 5

### Defining the analysis person-time and strata

Specifying the population and period for which estimates are sought, and the granularity with which these may be computed, determines most of the subsequent steps. In some scenarios, this will be straightforward (e.g. an entire country or a specific region is affected by armed conflict with a clear start and end date). In other cases, the analysis may be conducted to estimate mortality up to a certain time point in the crisis.

The definition of ‘crisis’ also needs to be made explicit: for example, Somalia has experienced 30 years of armed conflict; against this backdrop, drought and flooding emergencies have repeatedly occurred. Our analyses to date in Somalia have aimed to estimate mortality attributable to exceptional food insecurity events (2010–2012, 2017–2018) [20] triggered by drought, i.e. above and beyond any excess deaths caused by the protracted conflict alone. Accordingly, we have defined the period of analysis as that over which key food security indicators and other markers of crisis conditions were reported to be unusually poor. In Nigeria, we wished to estimate mortality attributable to the armed conflict between the government and Boko Haram, which affects three states (Borno, Yobe, Adamawa) in the northeast: this is a more straightforward scenario in which a relatively recent baseline of no conflict precedes the crisis. Refugees who leave the crisis-affected region should also be considered within the study population. However, this bears several complexities: for example, refugees will be exposed to different risk factors and may paradoxically experience lower mortality than if they had remained in their country of origin, implying a negative excess mortality: this has been documented for South Sudanese refugees in Uganda [21], and could plausibly apply to the large Syrian refugee population now living in Europe.

In practice, the person-time boundaries of the analysis and the smallest level of stratification ($kt$) may be constrained by data availability. However, if possible a ‘buffer’ period (e.g. 6–12 months pre-crisis) should be included in the analysis to allow exploration of lagged effects of predictors on mortality and to use ‘baseline’ observations to set counterfactual values for the predictors (see below, ‘Excess mortality estimation’ section). Furthermore, stratification should be as granular as possible to maximise observations available for model fitting and the utility of estimates.

As detailed below, sample surveys conducted by various humanitarian actors are the commonest source of mortality ground data with which to fit and validate models. In a Somalia study (2010–2012) [11] we conducted in the aftermath of a severe famine, nearly all such surveys had as their sampling universe the intersection of regional and livelihood zone boundaries: for example, within Gedo region some surveys were designed to represent communities that predominantly relied on pastoralism, while other surveys covered IDPs or riverine agriculturalists. Most of the predictors and demographic estimates were also collected at or could be aggregated to this stratification level, and by month. Our chosen $kt$ was thus regional livelihood zones and months (Table 2).

**Table 2:** Geographic analysis strata, Somalia (2010–2012) [11]

Region	Number of strata, by livelihood type					Total strata
	Pastoralist	Agro-pastoralist	Riverine	Urban	IDP	
Bakool	1	1	0	0	1	3
Banadir (Mogadishu)	0	0	0	1	1	2
Bay	1	1	0	1	1	4
Galgaduud	1	1	0	0	1	3
Gedo	1	1	1	0	1	4
Hiraan	1	1	1	0	1	4
Lower Juba	1	1	1	1	1	5
Middle Juba	1	1	1	0	1	4
Mudug	1	1	0	0	1	3
Lower Shabelle	1	1	1	1	1	5
Middle Shabelle	1	1	1	1	1	5
Totals	10	10	6	5	11	42

In more recent work, available data have supported stratification by level 2 administrative unit (counties and districts, respectively).

## Implementation of specific steps

### Data collection and management steps

#### Mortality data

Ground mortality observations are required to train and validate a predictive model.

The Standardised Monitoring and Assessment of Relief and Transitions (SMART) initiative [22] has developed a globally applicable protocol for rapid surveys that primarily aim to estimate the prevalence of acute malnutrition, but often also include a questionnaire module that elicits information from sampled households on their demographic experience over a retrospective ‘recall’ period, typically 3–6 months long [23]. SMART surveys are highly standardised and conducted routinely in most humanitarian responses [24], typically at administrative level 2 or similarly small scale. Surveys mostly rely on two-stage cluster sampling, though some, e.g. in IDP camps, are exhaustive or use systematic random selection. Sample sizes of 300–1000 households and 20–30 clusters are typical, i.e. sampled households are only a small fraction of the total. Survey design and analysis are automated by Emergency Nutrition Assessment (ENA) software, reducing the potential for surveyor error [25].

**Fig. 2:**
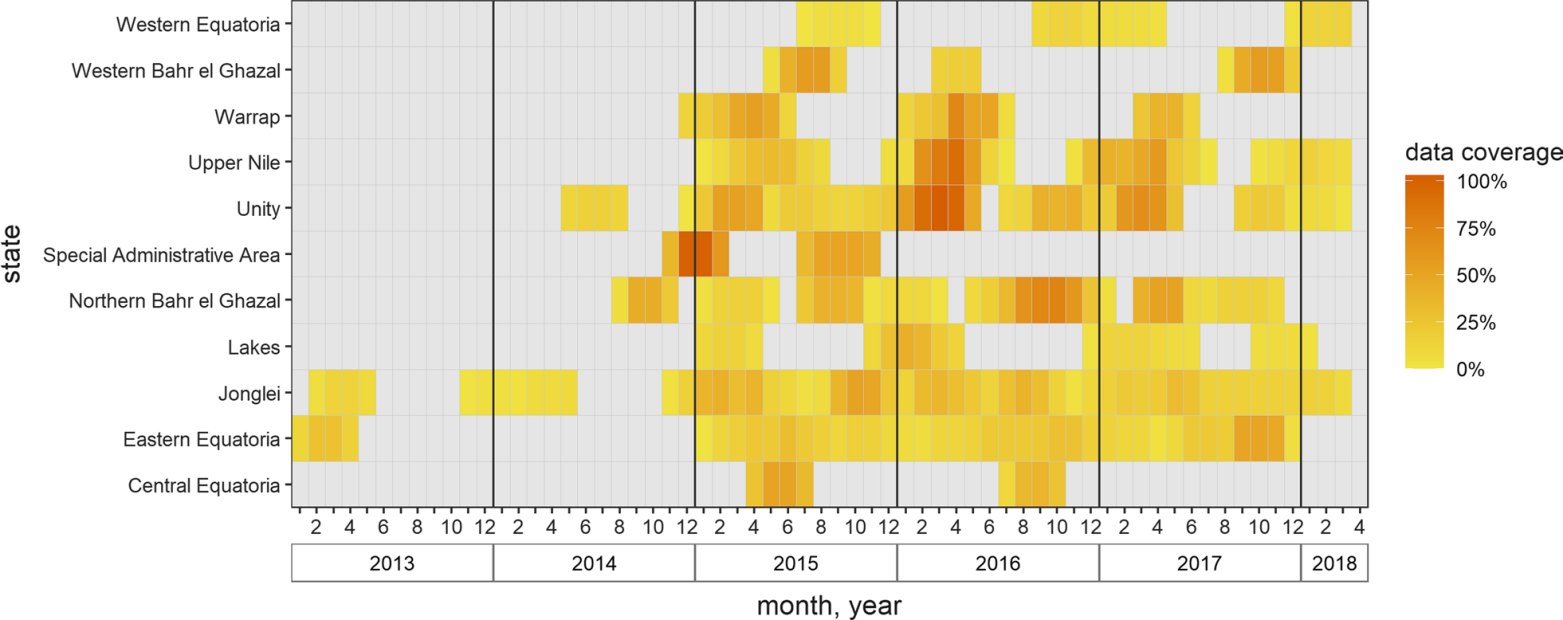
Coverage of SMART mortality surveys, by state and month, South Sudan, 2013–2018. Heat colours denote the percentage of the state’s population that fell within the sampling frame of at least one survey

We identified 205 analysis-eligible SMART surveys in Somalia (2010–2012), 210 in South Sudan (2013–2018), 91 in Somalia (2014–2018) and 70 in Nigeria (2016–2018). Despite these substantial numbers, geographic and period data coverage can be sparse, as illustrated in Fig. 2 for South Sudan.

**Fig. 3:**
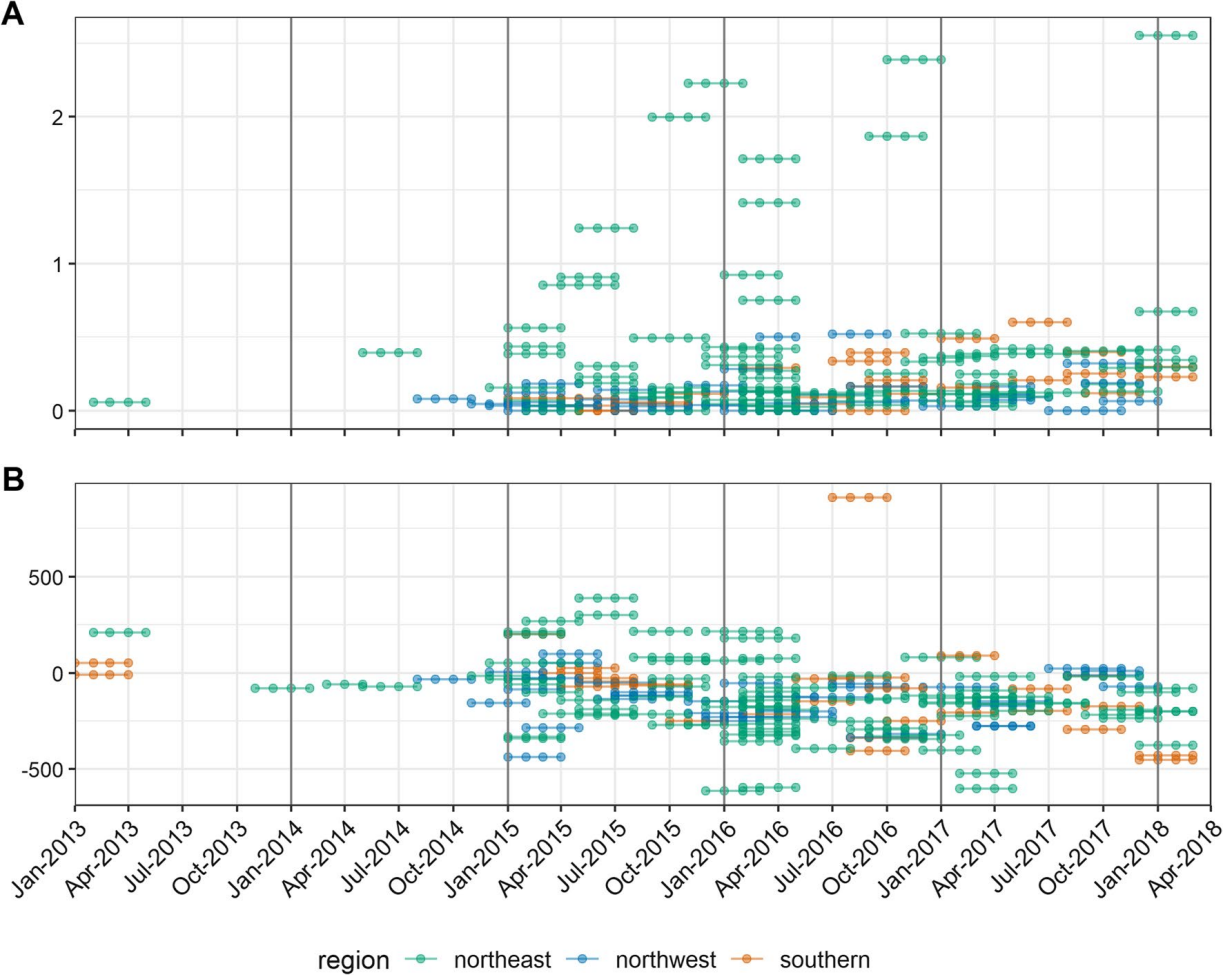
Trends in selected survey-estimated indicators, South Sudan, 2013–2018. Each dot-line segment denotes the recall period of one survey. Panel **A** death rate due to injury trauma per 10,000 person-days. Panel **B** net household migration rate per 1000 person-years

After cleaning datasets to resolve errors (e.g. values out of the allowed range), the crude death rate (CDR), under 5 years death rate (U5DR or CDR among the population aged under 5 years), crude birth rate, in-, out- and net migration rate, and, for individual questionnaire surveys only, cause- and gender-specific death rates may be computed (Additional file 1: page 2 and Table S1). The CDR and U5DR in particular are widely used by humanitarian actors to benchmark the severity of a crisis in health terms [1]. Inspection of crude patterns in survey indicators may be informative: for example, in South Sudan many surveys indicated high injury-attributable death rates and relative risks of dying among males, compared to females (Fig. 3).

Humanitarian surveys have varying robustness [26, 27]. While SMART survey reports do not systematically report quality issues, they should nonetheless be scrutinised to identify potential biases, particularly any restriction of the effective sampling frame to only a fraction of the intended sampling universe, due for example to insecurity or inaccessibility. We attribute to each survey $s$ a weight ${w}_{s}={{w}_{B,s}w}_{Q,s}$, where${w}_{B,s}$, a representativeness weight, is the approximate fraction of the sampling universe that was actually included in the sample, as per the survey’s report (for example, if a report states that the sampling frame excluded 3 out of 5 districts, we set ${w}_{B,s}=0.4$; where an unspecified number of sampling units are excluded from the sampling frame, we assume ${w}_{B,s}= 0.5$); and ${w}_{Q,s}$ is a quality weight derived from the dataset (see Additional file 1: page 3).

#### Predictor data

If the statistical objective of analysis is merely to predict the death rate, any set of predictor variables that does so accurately, whatever their causal relationship with mortality, may be appropriate. However, choosing predictors that are causally related to mortality, or proxies for mortality risk determinants, is likely to enhance predictive power and help assess the model’s internal validity. To this end, we have defined a generic framework of factors leading to crisis mortality (Additional file 1: Figure S1). At least some of the selected predictors should be related to plausible drivers of excess mortality risk: for example, in a drought-triggered food security crisis these might include rainfall, food purchasing power, burden of malnutrition and the incidence of epidemics (cholera, measles); in an armed conflict, the intensity of violence and disruptions to public health services might be more relevant. Identifying such ‘crisis-specific’ predictors is critical, as the method defines no-crisis scenarios by specifying counterfactual values for these very predictors.

In armed conflict settings and humanitarian responses, data collection is often unsystematic and disrupted [28]. In our experience to date, data are available for only few causal factors, and negotiation with agencies and humanitarian coordination mechanisms holding non-public datasets occupies a large share of analyst time. Such datasets generally have poor integrity; they are typically entered onto spreadsheet software without standardisation of geographical nomenclature, value cell or formula protections, variable dictionaries or automatic error checking—thus necessitating extensive curation. Missingness is a common problem (Additional file 1: Figures S2 and S3). We retain potential predictor datasets by applying a ‘70–70–70’ rule, namely ≥ 70% complete for ≥ 70% of $k$ and ≥ 70% of $t$. Remaining missingness is resolved through imputation, either statistical or manual (i.e. based on contextual knowledge). In order to reduce the influence of outliers (some of which may be data entry errors), where appropriate we apply moderate smoothing or running means to time series. Details of predictors considered are presented in crisis-specific papers; Table 3 shows predictors included in the final models for each of the crises studied thus far.

**Table 3:** Predictors included in the final models of CDR, by crisis

Domain in causal framework	Predictor	Crisis			
		Somalia (2010–2012)	South Sudan (2013–2018)	Somalia (2014–2018)	Nigeria (2016–2019)
	Region	X	X	X	
Exposure to armed attacks/insecurity	Incidence of armed conflict incidents	X	X	X	
Exposure to armed attacks/insecurity	Incidence of attacks against aid workers	Not available	X		
Food insecurity and livelihoods	Most prevalent livelihood type	X	X		X
Food insecurity and livelihoods	Terms of trade	X	X		
Food insecurity and livelihoods	Cereal staple price				X
Forced displacement	Proportion of the population that is internally displaced	Not available	X		
Nutritional status	Rate of admissions of severe malnutrition cases	Not available	Not available	X	
Burden of endemic infectious diseases	Health-facility based incidence of malaria	Not available		X	Not available
Epidemic occurrence and severity	Occurrence of epidemics	X (any epidemics)	X (cholera)	X (measles)	Not available
Humanitarian service functionality	Ratio of humanitarian actors to population				X
Humanitarian service functionality	Presence of food sector humanitarian assistance	X			
Humanitarian service coverage	Food distributed per capita		X		Not available
Health service coverage	Vaccination coverage	Not available	X	Not available	X

### Analysis steps

#### Predictive model fitting

If the raw datasets of mortality surveys are mostly unavailable, only stratum-level regression is feasible (Additional file 1: page 9). If raw data for most mortality surveys are available, household-level regression may be undertaken. SMART surveys do not report the exact date of deaths within the recall period: therefore, we merge predictor with survey data by computing the former’s weighted mean over the survey’s recall period. The data structure is partly longitudinal: for example, in Nigeria, five consecutive survey rounds took place during 2016–2019. While each survey round drew an independent sample, most Local Government Areas (LGAs; administrative level 2 units) hosted survey clusters during each round. In Somalia, some surveys were only representative of IDP settlements or urban areas within districts: we assume simplistically that district-wide predictor values also apply to these populations.

We use a generalised linear model with weights ${w}_{s}$ (see above) and a quasi-Poisson distributional assumption to account for overdispersion in the death count outcome. The model’s formula is thus:

2 $$\log d_{{i,j,k,T_{r,s} }} = x_{{1,k,T_{r,s} }} \beta_{1} + x_{{2,k,T_{r,s} }} \beta_{2} + x_{{3,k,T_{r,s} }} \beta_{3} \ldots + x_{{p,k,T_{r,s} }} \beta_{p} + u_{j} + u_{k} + \log \Pi_{{i,j,k,T_{r,s} }} + \epsilon_{i,j,k}$$

where ${d}_{i,j,k,{T}_{r,s}}$ is the number of deaths in household $i$ within survey cluster $j$ and geographic stratum $k$ occurring during the recall period ${T}_{r}$ of survey $s$, where $r$ means recall; ${x}_{1,k,{T}_{r,s}}, {x}_{2,k,{T}_{r,s}}, {x}_{3,k,{T}_{r,s}}\dots {x}_{p,k,{T}_{r,s}}$ are the values of predictors ${x}_{1}$,${x}_{2}$, ${x}_{3}\dots {x}_{p}$ averaged over the survey’s recall period, and for stratum$k$; ${\beta }_{1}$, ${\beta }_{2}$, ${\beta }_{3}\dots {\beta }_{p}$ etc., are the corresponding fixed-effect linear coefficients; ${u}_{j}$ and ${u}_{k}$ are, respectively, random effects for cluster $j$ and stratum $k$, assumed to follow a normal distribution with mean 0 (${u}_{j}\sim \mathcal{N}(0,{{\sigma }_{{u}_{j}}}^{2}$) and ${u}_{k}\sim \mathcal{N}(0,{{\sigma }_{{u}_{k}}}^{2}$)), and capturing a plausible hierarchy of data as well as the repeated nature of observations; $\mathrm{log}{\Pi }_{i,j,k,{T}_{r,s}}$ is an offset to account for varying household person-time $\Pi$ at risk (Additional file 1: Table S1); and $\epsilon_{i,j,k}$ is the residual error not explained by the model. We validate candidate models for out-of-sample prediction through k-fold cross-validation (CV; partition of data into folds is at the ${k,T}_{r,s}$ level given predictors are not specified below this level). We use the mean Dawid–Sebastiani score ($\mathrm{DSS}$) [29] as a proper scoring rule appropriate for count outcomes to evaluate model fit on the training data and on CV (in the latter case, we take the mean $\mathrm{DSS}$ across all folds). After exploratory analysis, where possible we select between maintaining the continuous version of the predictor or categorising into bins, as well as alternative lags, based on the lowest ${\mathrm{DSS}}_{\mathrm{CV}}$, and screen out predictors that are not significantly better-fitting than the null model based on an F-test p-value threshold. We fit each possible combination of remaining predictors ($X$predictors = ${2}^{X}$ possible combinations) and shortlist candidate models whose $\mathrm{DSS}$ is within a given bottom quantile. We select the final set of predictors based on ${\mathrm{DSS}}_{\mathrm{CV}}$, plausibility considerations and whether they are crisis-specific (see above). We test for plausible interactions and, lastly, add random effects, retaining the mixed model if its ${\mathrm{DSS}}_{\mathrm{CV}}$ improves on the fixed-effects alternative. In practice, a mixed model may be of limited utility if most prediction happens for person-time with new levels of the random effect (e.g. in geographic strata not covered by any survey used to train the model on).

**Fig. 4:**
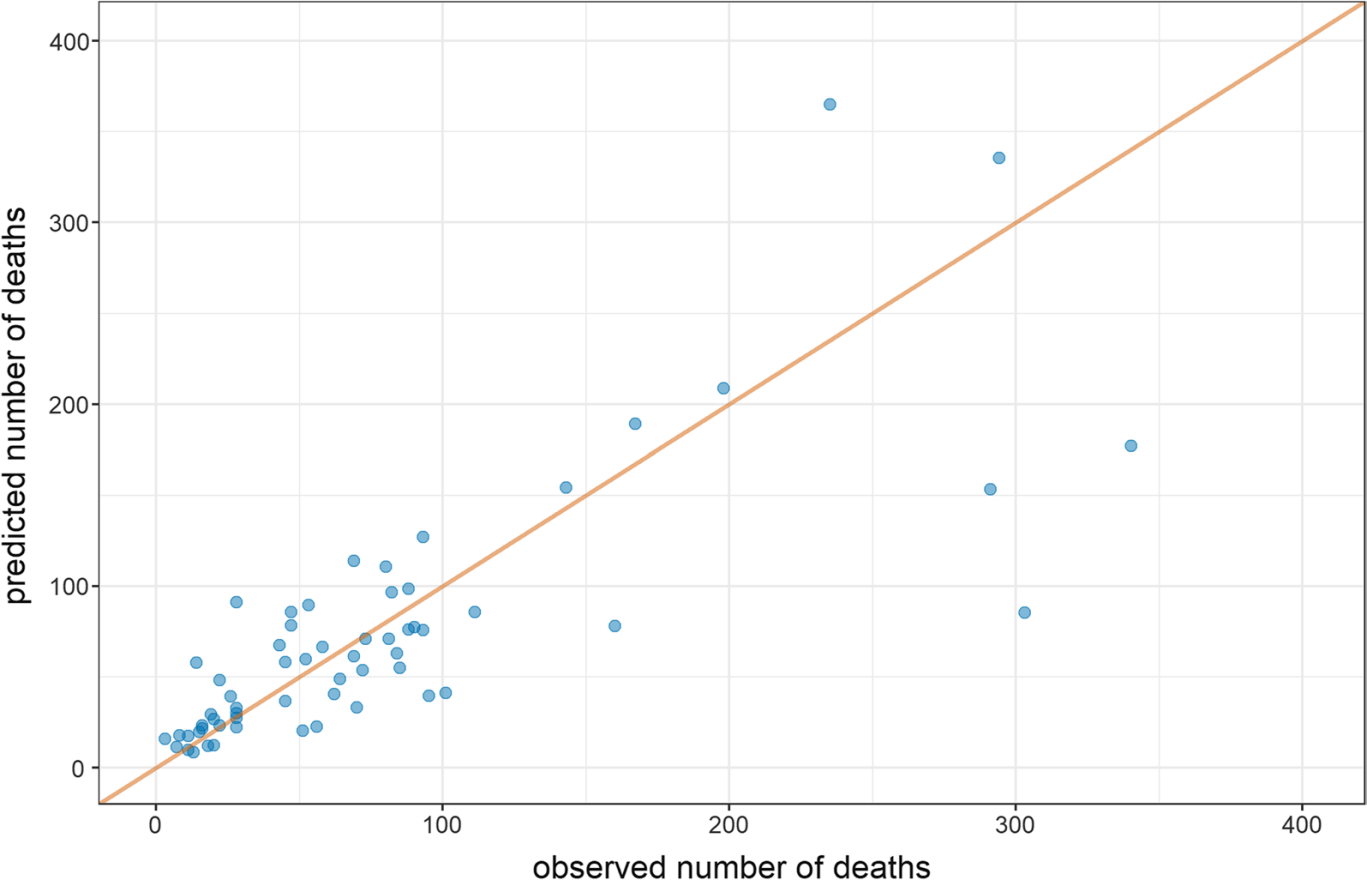
Predicted versus observed numbers of deaths per stratum (county), South Sudan, 2013–2018, based on ten-fold cross-validation. The red line indicates perfect fit

As an example, we provide in Table 4 model coefficients and performance metrics for South Sudan, all computed based on observations and predictions aggregated at the ${k,T}_{r,s}$ level; predictive accuracy on cross-validation is shown in Fig. 4. As shown, the DSS, which, like other prediction scores, quantifies the error between observations and predictions, increases only slightly on CV, indicating that the model only marginally overfits data and is valid when used out-of-sample. There is also little evidence of predictive bias. Aside from moderately good performance, model coefficients support model validity: mortality increases with insecurity and where measles epidemics are present, but decreases if people are living in Protection of Civilians camps (in South Sudan, these places afforded relative safety and more intense humanitarian services) and as purchasing power improves.

**Table 4:** Final model to predict crude death rate, South Sudan (2013–2018)

Fixed effect	Relative rate	95% CI	*p*-value
Intercept	0.00014	0.00008 to 0.00022	< 0.001
*Region*			
Northeast	[Ref.]		
Northwest	0.54	0.41 to 0.72	< 0.001
Southern	0.80	0.51 to 1.25	0.326
*Main livelihood type*			
Agriculturalists	[Ref.]		
Agro-pastoralists	0.82	0.55 to 1.22	0.329
Pastoralists	1.24	0.69 to 2.23	0.478
Displaced to Protection of Civilians camps	0.52	0.34 to 0.81	0.004
*Rate of insecurity events (per 100,000 people per month, lag* = *4 months)*			
0	[Ref.]		
0.01 to 0.99	1.16	1.02 to 1.32	0.021
≥ 1.00	1.32	1.08 to 1.62	0.008
*Uptake of measles vaccine (doses administered per 100,000 people per month)*			
0	[Ref.]		
0.1 to 199.9	0.83	0.69 to 0.99	0.042
200.0 to 399.9	0.76	0.60 to 0.97	0.025
≥ 400.0	0.56	0.43 to 0.74	< 0.001
*Terms of trade purchasing power index (Kg of white wheat flour that an average goat can be exchanged for; 3 months running average, lag = 3 months)*	0.992	0.987 to 0.996	< 0.001
*Rate of violent incidents affecting humanitarian staff (per 100,000 per month, lag* = *4 months)*			
0	[Ref.]		
≥ 0	1.19	1.04 to 1.36	0.010
*Incidence rate of confirmed or probable measles cases (per 100,000 per month)*			
0	[Ref.]		
≥ 0	1.30	1.15 to 1.47	< 0.001

Model performance metric	Value	Notes
Dawid–Sebastiani score (internal prediction)	26.9	$\frac{{\left( {{\text{observed }} - {\text{predicted}}} \right)^{2} }}{{{\text{variance}}}} + 2 \times \log \left( {{\text{variance}}} \right)$
Dawid–Sebastiani score (out-of-sample prediction)	29.2	Based on tenfold cross-validation (CV)
Relative bias (on CV)	− 0.064	$\frac{{{\text{predicted}} - {\text{observed}}}}{{{\text{observed}}}}$
Relative 95% precision (mean across strata on CV)	1.011	$\frac{{0.5 \times \left( {{\text{upper}} 95\% {\text{CI}} - {\text{lower}} 95\% CI} \right)}}{{{\text{predicted}}}}$
Coverage of 80% confidence intervals (on CV)	0.754	Proportion of stratum observations falling within the confidence interval of the prediction
Coverage of 95% confidence intervals (on CV)	0.901	

Note that the predictors and values below differ from the original model presented in the study report, as they arise from an improved fitting procedure. Random effects are omitted

#### Excess mortality estimation

In our framework, excess mortality estimation requires projecting the death toll in counterfactual no-crisis scenarios. These scenarios should specify counterfactual values for all crisis-specific predictors included in the final models, and for the population denominators. Several approaches to set counterfactual values may be used: (i) in the absence of a crisis, it may be assumed that certain predictors or types of displacement would have taken a zero value: for example, epidemics (e.g. cholera, measles) that are known to be associated with extreme food insecurity crises might not have occurred; similarly, no war-related displacement would have happened; (ii) pre-crisis values of the predictors, if available, may be adopted as counterfactuals: for some predictors (e.g. market prices), we use the local average (e.g. the district median prior to the crisis’ start); for others (e.g. rainfall), seasonality should also be considered; (iii) if no pre-crisis data are available, levels from reasonably comparable regions within the country that are not affected by the crisis may instead be considered. Table 5 shows ‘most likely’ counterfactual assumptions for the South Sudan analysis we previously conducted. To explore uncertainty in these assumptions, we also define reasonable best- and worst-case scenarios.

**Table 5:** Most likely scenario counterfactual assumptions, South Sudan (2013–2018)

Variable	Counterfactual assumptions	Notes
Proportion of IDPs	The proportion of IDPs in each county would have been equal to the mean total across South Sudan in Jan 2012–Nov 2013, multiplied by the county’s mean percent share of total IDPs during Dec 2013–Apr 2018	Assume that the relative scale of internal displacement during the war reflects each county’s general potential for displacement Accordingly, in the counterfactual denominator IDPs are ‘returned’ to their counties of origin pro rata to the assumption
	Same number of IDPs in Pibor county as mean of 2012–2013	Assume conflict in Pibor County would have continued, as it pre-dated the current civil war
Incidence of armed conflict events	Mean of 2012–2013 level within each county, or actual level, whichever is lower	Pre-crisis baseline
Incidence of attacks against aid workers	Mean of 2012–2013 level within each county, or actual level, whichever is lower	Pre-crisis baseline
Terms of trade purchasing power index	Mean of 2012–2013 levels per state	Pre-crisis baseline
Uptake of measles routine vaccination	On an annual basis, no lower than the mean of 2012–2013 levels per county	Assumption preserves any improvements in vaccination coverage observed during the crisis period in any county
Measles incidence	Mean of 2012–2013 level within each county, or actual level, whichever is lower	Pre-crisis baseline

To propagate error in the model predictions of ${y}_{A,k,t}$ and ${y}_{C,k,t}$ into final estimates, we can set up a bootstrap simulation that, for a large number of iterations and each $kt$ stratum, implements Eq. (1) by drawing random values from the models’ normal distribution of log standard errors. Outputs of each iteration are then summed across all $kt$ or for specific aggregations of interest (e.g. a single year within the crisis period), and point estimates and 95% confidence intervals are computed as the median, 2.5th and 97.5th percentiles of the resulting distribution of iteration sums. Note that if counterfactual population denominators are considerably different from the actuals (e.g. if large-scale displacement outside the region of interest has occurred), comparing actual and counterfactual mortality is fraught due to the difference in at-risk populations: we therefore scale excess death rates to the actual population denominators.

#### Sensitivity analyses

While a number of sensitivity analyses may be conducted to explore estimate uncertainty, we focus here on two particularly important issues.

##### Population denominator uncertainty

Most displacement data in crisis settings do not arise from statistically robust estimation methods. Over-reporting of population figures may occur if population counts are perceived as registration for relief allocation [30]. Conversely, insecurity and lack of connectivity may result in undetected population movements. We thus explore combinations of sensitivity values for both displacement and demographic estimates (as a ratio of true to reported values, where values < 1 indicate over-reporting, and vice versa), and re-run analysis accordingly.

##### Under-estimation of mortality in surveys

In previous South Sudan work, possible under-estimation of deaths among children under 5 years has been noted, as indicated by a low ratio of under 5 years to all-age deaths and low proportion of infant deaths (Table 6). Similar concerns have been raised in Yemen [31]. Under-reporting of infant and particularly neonatal deaths is plausible, due to stigma and/or emotional trauma associated with losing a young child or insufficient probing during questionnaire administration. We thus re-run analysis after augmenting the model training data (number of deaths and person-time within surveyed households) based on a varying assumed proportion of all deaths that are unobserved (Additional file 1: page 10).

**Table 6:** Average survey-estimated crude death rate per 10,000 person-days, under 5 years death rate per 10,000 person-days and percentage of infant deaths among all deaths below 5 years of age, by country

Characteristic	Nigeria (2016–2018)	Somalia (2014–2018)	South Sudan (2012–2018)
Eligible surveys (N)	70	97	181
Crude death rate	0.55 (0.17 to 1.58, 70)	0.43 (0.00 to 1.61, 97)	0.67 (0.04 to 4.22, 181)
Under 5 years death rate	1.14 (0.23 to 4.46, 70)	0.66 (0.00 to 2.48, 97)	0.72 (0.00 to 3.94, 181)
Percentage of < 5 years old deaths that were among infants < 1 year old	35% (0% to 100%, 70)	43% (0% to 100%, 59)	33% (0% to 100%, 145)

Numbers are the median of point estimates among available surveys, and, in parenthesis, the range of point estimates and number of surveys the statistics are based on

## Discussion

### Advantages of the method

The approach we have described can efficiently reconstruct the evolution of mortality across long retrospective periods and large areas, including where ground data collection would be unfeasible due to inaccessibility or the difficulty of asking households to recall events over a long recall period; in South Sudan, a setting with virtually no vital events registration, our application of the method generated evidence supporting a large excess death toll (about 380,000, half attributable to intentional injuries) attributable to 5 years of war, that might otherwise have evaded historical documentation forever. Somalia estimates (2010–2012) documented the impact of one of the worst famines in the past decades. Predictive models underlying the estimates have quantifiable external validity. While predictive power is ultimately their most important attribute, observing the directionality of coefficients can help to appraise internal validity, particularly if dose–response associations are noted. To our knowledge, no other studies have developed statistical models that predict with reasonable accuracy the crude or under 5 years death rate among some of the world’s most vulnerable populations. A known challenge of crisis-attributable mortality estimation is defining an appropriate counter-factual: our method achieves this by generating non-crisis death tolls through the same statistical processes that result in the estimate of actual mortality, yielding meaningful confidence intervals. It explicitly links the definition of the crisis with the choice of counterfactual predictors and values, drawing upon a causal framework of how excess mortality comes about and contextual understanding of the crisis itself. Lastly, the method does not require any primary data collection.

### Known limitations

The method’s main limitations reflect sources of unknown error in input data: (i) error in the predictor data, for example arising from differences in the way predictors are measured over time or in different locations; random error would result in underestimation of associations between predictors and mortality, or ‘regression dilution’ in predictive terms; bias could cause over- or underestimation; (ii) bias in mortality data, e.g. due to problems with under-ascertainment of deaths (see above), which survey quality weights may reduce but not eliminate; (iii) nonparametric uncertainty around population and displacement estimates; (iv) demographic projections based on inaccurate assumed growth rates (both (iii) and (iv) will be discussed in a separate paper); (v) inappropriate assumptions on counterfactual conditions; and (vi) omission of excess mortality among people who migrate out of the affected region (e.g. refugees), or due to long-term impacts of the crisis beyond its resolution. These limitations imply that estimates should be interpreted with caution, with reference to confidence intervals and after thorough exploration of uncertainty through alternative counterfactual scenarios and sensitivity analyses.

Perhaps the most important limitation among the above concerns how counterfactual conditions are specified. Varying predictor values to represent no-crisis conditions presents analogies with both interrupted time series [32] and growth models [33]. However, our approach quantifies the effects of multi-factorial and dynamic crises rather than a single public health intervention implemented in a fairly stable setting: as such, our estimates rely heavily on a few model predictors faithfully representing a more complex system; moreover, counterfactual values for many predictors (e.g. food security, vaccination coverage) are not simply zero, as in the case of a counterfactually absent intervention, but rather some quantity relative to the actual levels.

### Data requirements

The method’s applicability is limited by the following data requirements: (i) at least *some* ground mortality information arising from a population-based method of recognised validity, e.g. a survey or prospective surveillance system. Such data should be granular in nature, i.e. representative of small geographic units and time periods (alternatively, one could use large-area surveys as long as the location of surveyed communities is reported in the dataset). Some documentation (e.g. survey reports) should be available to scrutinise methods; (ii) data covering the entirety or most of the person-time of interest for at least a few variables that may plausibly be expected to predict mortality. The system for measuring these predictors should have remained consistent over time. The pattern of data missingness should be mostly random: missingness clustered in specific areas or periods (particularly at the start or end of the time series, or where mortality data are also least available) makes imputation harder and more bias-prone; (iii) reasonable demographic estimates based on a census or similarly robust data collection exercise, performed no more than a few years prior to the analysis; in addition, data on displacement (including both the geographic unit of origin and that of arrival) covering most or all of the person-time should be available, or composable from existing reports and databases.

Minimal data requirements, e.g. how many ground surveys or predictor variables are needed, are difficult to establish a priori: the predictive power of the model is a function not just of the amount of data, but also of the extent to which these data capture population variability and the local strength of correlation between predictors and mortality. As such, an additional limitation of the method is that the precision, and thus interpretability, of estimates arising from it may only become clear a posteriori.

### Computational implementation

With the exception of step 2 (population denominator reconstruction), for which only crisis-specific analysis methods appear feasible, we have developed generic R analysis scripts that implement estimation steps for any crisis setting and generate output datasets, tables and graphs (see Additional file 1: pages 10–13 and https://github.com/francescochecchi/mortality_small_area_estimation). The analyst interacts with these scripts through Microsoft Excel spreadsheets containing input datasets and various parameters to control the analysis.

## Conclusions

We are currently testing an extension of the method for forecasting mortality over short time horizons of 3–6 months: this could provide an efficient means to do real-time estimation across the crisis-affected region, thereby generating information for decision-makers tasked with allocating humanitarian resources. Key requirements for such an application would be immediate predictor data sharing and standing capacity to implement analysis.

Other improvements to the method are worth exploring. As instances of its use accumulate, a Bayesian estimation framework specifying informative priors for key predictor coefficients (e.g. armed conflict intensity) may be attractive. Improvements to model fitting could include machine learning techniques or Bayesian model averaging; due to limited resources, we have not systematically compared our generalised linear model with any of these alternatives. Indeed, these further developments will require dedicated scientific resources and buy-in from humanitarian stakeholders who hold access to key input data.

*Disclaimer *Geographical names and boundaries presented in this paper are used solely for the purpose of producing scientific estimates, and do not necessarily represent the views or official positions of the authors, the London School of Hygiene and Tropical Medicine, any of the agencies that have supplied data for this analysis, or the donors. The authors are solely responsible for the analyses presented here, and acknowledgment of data sources does not imply that the agencies or individuals providing data endorse the results of the analysis.

## Supplementary Information

**Additional file 1:** Additional details on specific analysis steps.

## Data Availability

The data that support the findings of this study are available from various United Nations and non-governmental agencies, but restrictions apply to the availability of these data, which were used under license for the current study, and so are not all publicly available. Data are however available from the authors upon reasonable request and with permission of the above agencies. Furthermore, we have uploaded curated R scripts and complete datasets for Somalia on https://github.com/francescochecchi/mortality_small_area_estimation (also see Additional file 1: pages 10–13). These materials should enable independent replication of all our analysis steps. Data will be made available to the extent possible as part of the publication of country-specific papers.

## Abbreviations

CDR: Crude death rate
CV: Cross-validation
DSS: Dawid–Sebastiani score
ENA: Emergency Nutrition Assessment
IDP: Internally displaced person
LGA: Local Government Area (Nigeria)
SMART: Standardised Monitoring of Relief and Transitions initiative
U5DR: Under 5 years death rate
