# Supplementary material for: A method for small-area estimation of population mortality in settings affected by crises
Source: Popul Health Metr. 2022 Jan 11;20:4. doi: 10.1186/s12963-022-00283-6 (PMC8751462; doi:10.1186/s12963-022-00283-6)
Supplement: Supplementary file 1 — Additional file 1: Additional details on specific analysis steps. [file 12963_2022_283_MOESM1_ESM.docx]

A method for small-area estimation of population mortality in settings affected by crises

ADDITIONAL FILE

Francesco Checchi PhD*

Adrienne Testa MSc

Amy Gimma MSc

Emilie Koum-Besson MSc

Abdihamid Warsame MSc

Department of Infectious Disease Epidemiology

Faculty of Epidemiology and Population Health

London School of Hygiene and Tropical Medicine

* Corresponding author: [Francesco.checchi@lshtm.ac.uk](mailto:Francesco.checchi@lshtm.ac.uk)

Table of contents

[Data collection and management steps 2](#_Toc82789612)

[Mortality data 2](#_Toc82789613)

[Aggregate versus individual mortality questionnaires 2](#_Toc82789614)

[Treatment of mortality surveys without available datasets 3](#_Toc82789615)

[Details on the SMART ENA anthropometric data quality score 3](#_Toc82789616)

[Alternative survey quality scoring checklist 3](#_Toc82789617)

[Predictor data 4](#_Toc82789618)

[Mortality causal framework 4](#_Toc82789619)

[Missingness 5](#_Toc82789620)

[Analysis steps 9](#_Toc82789621)

[Stratum-level regression as an alternative 9](#_Toc82789622)

[Comparison of the two approaches 9](#_Toc82789623)

[Sensitivity analysis: Under-estimation of mortality in surveys 10](#_Toc82789624)

[R analysis scripts and input datasets 10](#_Toc82789625)

[Description of the scripts 10](#_Toc82789626)

[Input datasets 10](#_Toc82789627)

# Data collection and management t steps

## Mortality data

### Aggregate versus individual mortality questionnaires

Table S1 compares the two types of mortality questionnaire instruments used by SMART surveys, and resulting calculations required to compute person-time and thus estimate death rates. The individual questionnaire is recommended and increasingly utilised.

Table S1. Comparison of aggregate and individual mortality questionnaires.

| Characteristic | “Aggregate” questionnaire | “Individual” questionnaire |
| --- | --- | --- |
| Questionnaire process | 1. Ask for the number of people $b$ who slept in the household during the previous night. 2. Ask for the number of people $j$ who joined the household during the recall period. 3. Ask for the number of people $l$ who left the household during the recall period. 4. Ask for the number of births $b$ during the recall period. 5. Ask for the number of deaths $d$ during the recall period. | 1. List people who slept in the household during the previous night: establish whether they joined or were born during the recall period. 2. List people who left during the recall period. 3. List people who have died during the recall period: establish whether they were born during the period (infant deaths) and ask about cause and location of death. |
| Age information | Under 5y, older | Individual ages in years |
| Gender information | No | Yes |
| Cause of death information | No | Yes, as reported by household next-of-kin. Categories vary by survey, but most surveys include ‘injury/trauma’, ‘disease’ and ‘other’ categories, and some distinguish ‘violence/killing’ from other injuries. |
| Calculation of person-time denominator (all rates except U5DR) | $\left( n_{\text{i}}+0.5d_{\text{i}}+0.5l_{\text{i}}- 0.5b_{\text{i}}- 0.5j_{\text{i}} \right)T_{r}$  where $n$ = people in household $i$ now; $d$ = dead; $l$ = left; $b$ = born; $j$ = joined; $T_{r}$ = recall period of the survey in days.  This expression assumes people joined and left households, were born and died at the mid-point of the recall period. | Same as for aggregate questionnaire. |
| Calculation of person-time denominator (U5DR) | $\left( n_{\text{i,u5}}+0.5d_{\text{i,u5}}+0.5l_{\text{i,u5}}- 0.5b_{\text{i,u5}}- 0.5j_{\text{i,u5}} \right)T_{r}$ ,  where $u5$ = under 5y. This expression assumes children joined and left households, were born and died at the mid-point of the recall period. Note that a more complete expression would also take into account aging out of the under 5 year group during the recall period, i.e. a term $+a_{\text{i,u5}}T_{r}$ should also be added, where $a$ = aged out of under 5y cohort. However, as individuals’ ages are not recorded in this questionnaire, the rate of aging out would have to be estimated from secular trends in the birth and death rates prior to the survey – this in turn entails several assumptions at this stage of the estimation process, for what is likely to be only a minimal reduction in bias (e.g. during a recall period of 3 months, assuming an equal proportion of under 5y olds per year cohort, only 5% of children would age out of the under 5y group). | $\left( n_{\text{i,u5}}+0.5d_{\text{i,u5}}+0.5l_{\text{i,u5}}- 0.5b_{\text{i,u5}}- 0.5j_{\text{i,u5}} \right)T_{r}+\left( n_{\text{i,5}}+0.5d_{\text{i,5}}+0.5l_{\text{i,5}}- 0.5j_{\text{i,5}} \right)\frac{T_{r}}{365}$ ,  Where $u5$ = under 5y and $5$ = 5y (60-71 months) old. The latter term is $a$, i.e. children who must have aged out of the cohort during the recall period, assuming age recall is accurate. Note that this aging out estimate assumes an equal distribution of demographic events across the 5y age bracket. |

We use the survey package in R software to calculate point estimates and 95%CIs for each indicator, assuming a Poisson distribution of deaths and adjusting standard errors for cluster sampling design. We then check that these correspond to the estimates in the survey report.

### Treatment of mortality surveys without available datasets

If no dataset is available for most surveys in a crisis, analysis may need to be done using the survey-level estimates (see below), as reported in available documents. For any such survey $s$, the standard error ($SE$) of log death rate may be estimated from the reported point estimate $\hat{y}_{s}$ and its 95%CI: since $y_{s,0.975}=e^{(ln \hat{y}_{s}+1.96SE(ln \hat{y}_{s}))}$ (and $y_{s,0.025}=e^{(ln \hat{y}_{s}-1.96SE(ln \hat{y}_{s}))}$), by rearrangement ${SE(ln \hat{y})}_{s}=\frac{\ln y_{s,0.975}- ln \hat{y}_{s}}{1.96}$ . The plausibility of reported 95%CIs can be verified by computing the asymmetry between the upper and lower CI: for any survey with asymmetric or nonreported CI, a 95%CI may be approximated based on the reported number of deaths under 5y and an assumed cluster sampling design effect $DEFF$ (${SE(ln \hat{y})}_{s}=\frac{1}{\sqrt{d_{s}}}\text{DEFF}\text{ )}$. A $DEFF$ of 2.0 is reasonably typical of crisis settings.

Whenever the proportion of deaths due to injury and/or violence are provided in the report, they can be multiplied by the reported $\hat{y}_{s}$ to compute the point estimate of the injury and/or violence death rates, and corresponding $SE$ as above. Any exhaustive surveys may be treated as simple random samples.

### Details on the SMART ENA anthropometric data quality score

The overall quality score produced by the ENA plausibility check report ($w_{A,s}$) is a composite of the following factors:

- - - the proportion of outliers (any extreme value is “flagged”);
    - the sex and age (6-29mo vs. 30-59mo) ratios;
    - the digit preference score for weight, height and middle-upper arm circumference measurements;
    - the mean and standard deviation of the weight-for-height distribution;
    - the skewness and kurtosis of the weight-for-height distribution;
    - the degree of overdispersion of the weight-for-height distribution.

The overall score is then categorised as follows: 0-9% is excellent, 10-14% good, 15-24% acceptable and ≥ 25% problematic.

To our knowledge no corresponding quality score has been proposed by the SMART Initiative for mortality data. We assume that, as the same team of surveyors collects anthropometric and mortality data, it is plausible that the quality of the two datasets would be correlated, and thus apply $w_{A,s}$ to mortality analysis.

In order to compute $w_{Q,s}$, we rescaled $w_{A,s}$ to unity by dividing it by the maximum across all surveys and took its complement, i.e. $w_{Q,s}=1-\frac{w_{A,s}}{\max_{s}\left\{ w_{A,s} \right\}}$ .

### Alternative survey quality scoring checklist

As an alternative to the above, we have developed a checklist (Table S2), adapted from Prudhon et al. (2011)^[[1]](#footnote-1)^, and based on detailed perusal of the survey report, if available.

Table S2. Survey quality checklist.

| Section and criteria | Score |
| --- | --- |
| 1. Sampling frame |  |
| - 1. Is the sampling universe defined? |  |
| - 1. Is the population size current and cross-checked? |  |
| 1. Sampling design |  |
| - 1. Is there a clear description of the sampling methodology? |  |
| - 1. Is sampling fully non-purposive (at every stage)? |  |
| - 1. IF systematic random sampling: is there a clear description of a non-arbitrary  sampling step? |  |
| - 1. IF cluster sampling: is there a clear description of cluster sampling? |  |
| 1. Cluster sampling – Household selection (*only if cluster sampling was done*) |  |
| - 1. Is there a clear description of the household selection? |  |
| - 1. IF segmentation was performed: was it done non-purposively and weighted? |  |
| - 1. IF first household selected randomly: Was the method by which the first household was sampled clear and non-purposive? |  |
| - 1. IF all households selected randomly: Was the method by which the first household was sampled clear and non-purposive? |  |
| - 1. IF all households were selected randomly: Was the source of lists current and cross-checked? |  |
| 1. Survey non-response |  |
| - 1. Is there a description of a valid revisit strategy? |  |
| - 1. Was the number of non-responding households reported? |  |
| - 1. Was the percentage of non-respondents < 15%? |  |
| 1. Precision of cluster sampling (*only if cluster sampling was done*) |  |
| - 1. Is there a sufficiently large (>25) number of clusters per explicit stratum or entire sampling frame? |  |
| 1. Questionnaire |  |
| - 1. Was there was a pre-piloted structured questionnaire in the local language? |  |
| - 1. Is the recall period clearly marked? |  |
| - 1. Was a calendar used as an aide-memoire? |  |
| - 1. Was the questionnaire for individuals only? |  |
| 1. Training & supervision |  |
| - 1. Were interviewers both trained and supervised? |  |
| - 1. Were surveys checked at the end of each day? |  |
| 1. Response bias |  |
| - 1. Was a recall period given in the analysis? |  |
| - 1. Was the recall period shorter than 2 years? |  |
| 1. Stratification (*only if explicit stratification was done*) |  |
| - 1. Was the analysis was weighted for unequal sampling? |  |
| - 1. Was the analysis stratified? |  |
| 1. Calculation |  |
| - 1. Was a sample size given? |  |
| - 1. Were CIs reported? |  |
| - 1. Was adjustment for clustering performed on the final effect estimate? |  |
|  |  |
| Overall quality score $\boldsymbol{w}_{\boldsymbol{Q,s}}$ = sum of average scores within each section (out of all applicable questions/criteria) / number of applicable sections (3, 5 and 9 depend on the survey’s design). Each question is scored 1 (yes) or 0 (no or unclear). |  |

## Predictor data

### Mortality causal framework

Figure S1 shows a proposed general framework of factors leading to (excess) mortality in crisis settings. Predictor variables that capture one or more factors in the framework are sought. Note that any predictor variables that are a function of population size (e.g. incidence of disease, doses of vaccine given) should first be divided by the best estimate of population for the corresponding stratum-month to construct population rates.

## Missingness

Figure S2 and Figure S3 show the completeness of predictor data for Somalia (2014-2018) in the district and time dimensions, respectively.

Physical health

Reproductive and neonatal health

Burden of NCDs

Burden of endemic infectious diseases

Epidemic occurrence and severity

**Trauma injuries** (intentional and unintentional)

Exposure to armed attacks/ insecurity

Forced displacement

Interruption of chronic treatment (HIV, TB, NCDs)

Addiction

Excess

Baseline

Averted

**Death rate**

Sexual and gender-based violence

Food insecurity and livelihoods

Feeding and care practices

Nutritional status

Humanitarian (public health) services

Service functionality

Service coverage

Service quality

Mental health and psychosocial functioning

Climate

Figure S1. Causal framework of predictors of excess mortality.


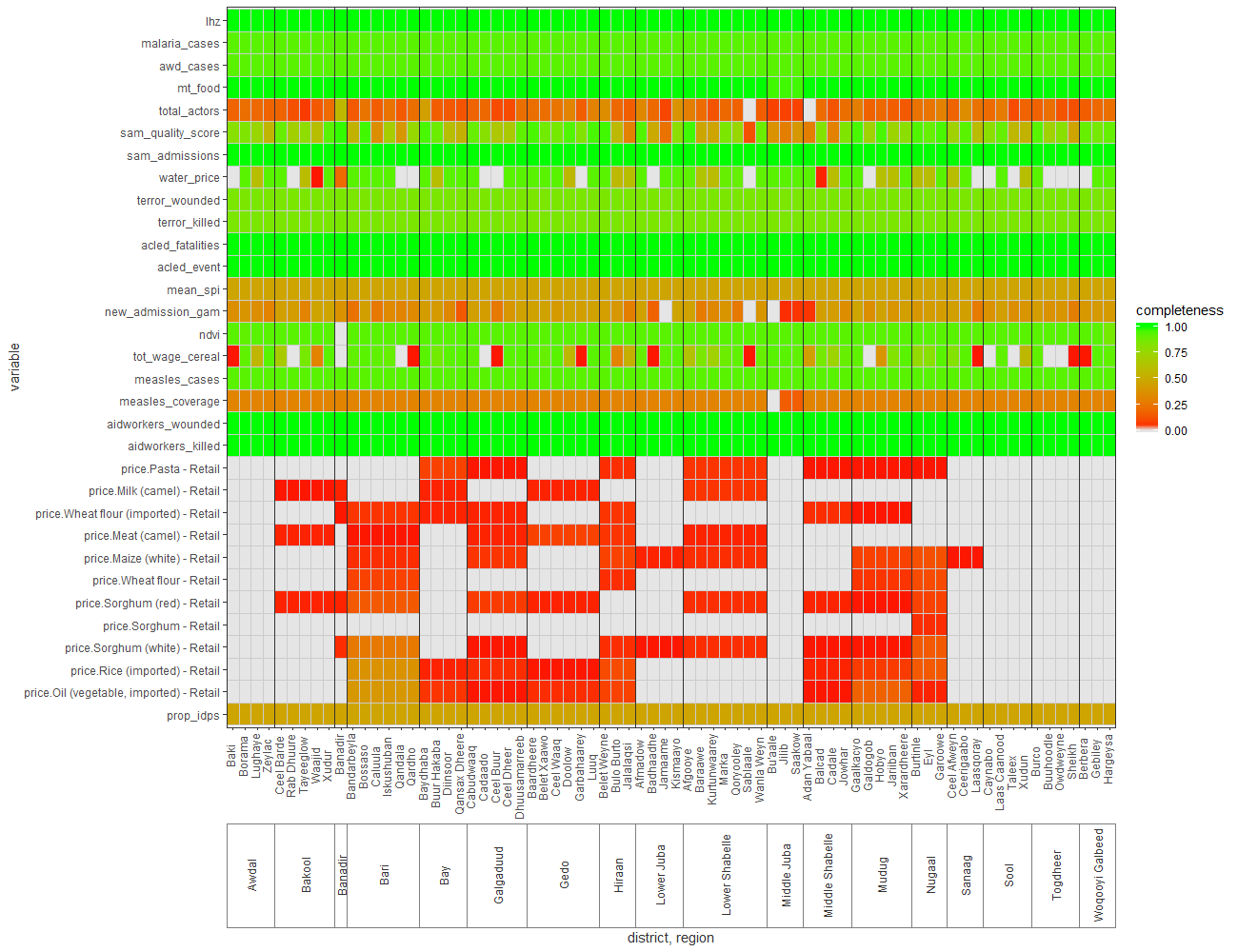


Figure S2. Completeness of predictor data by district, Somalia (2014-2018).


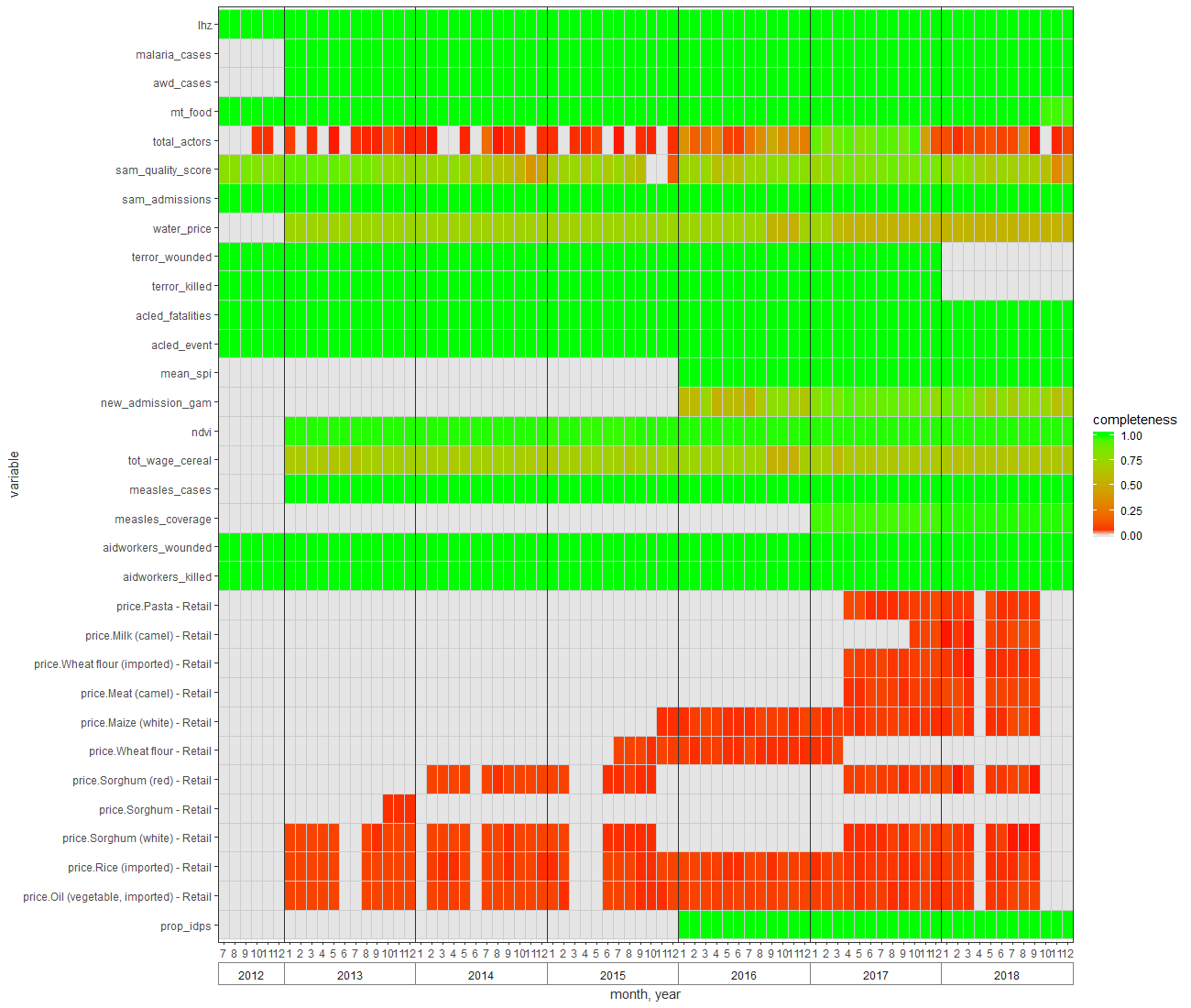


Figure S3. Completeness of predictor data by month, Somalia (2014-2018).

# Analysis steps

## Stratum-level regression as an alternative

As an alternative to household-level regression, particularly if raw datasets of mortality surveys are mostly unavailable, we can ignore data levels below $k$ and work with stratum-level survey metadata, which, on the log scale, consist of a point estimate $\ln\hat{y}_{s}$ and a normally distributed error distribution with standard deviation given by ${SE(ln \hat{y})}_{s}$ (see above for estimating the latter from 95%CIs). As long as the sampling universe of surveys is $k$, the model form is

Eq. 12 $y_{k}=x_{1,k}\beta_{1}+x_{2,k}\beta_{2}+x_{2,k}\beta_{2}\ldots+u_{k}+\epsilon_{k}$

where $y_{k}\sim e^{\mathcal{N(}\ln\hat{y}_{k},{{SE(ln \hat{y})}_{k}}^{2})}$ , i.e. a random variable drawn from the normal error distribution of the log death rate, back-transformed into the linear scale.

So as to adequately propagate survey estimate error into model coefficients, we can fit the model on a large simulated dataset of $Z$ random values of each $y_{k}$ . We have not yet established a robust $Z_{\min}$ empirically, but this could be done by fitting different models to simulated datasets of exponentially increasing size, until model error becomes stable. We think that one drawback of this data augmentation solution is that degrees of freedom will be grossly overestimated.

As stratum-level death rate estimates are continuous, an ordinary least-squares (OLS) model of the log-transformed data may be appropriate, though non-parametric alternatives may also be considered. Model selection and validation then follow the same steps as above, with the following variations:

- The adjusted $R^{2}$ could also be considered among the statistics of model fit;
- Having selected a final model, a Box-Cox transformation to the dependent variable can be tested, as opposed to a logarithmic or other transformation; Box-Cox transformations can only be parameterised in a model with co-variates, but may further improve model fit and conformance to OLS assumptions;
- If an OLS is used, standard diagnostics including heteroskedasticity, distribution and quantile-quantile plots of residuals are particularly important to verify.

### Comparison of the two approaches

Table S3 summarises expected advantages and drawbacks of the household- and stratum-level approaches. We have not compared the two in the same settings, and their relative predictive accuracy is thus unclear.

Table S3. Comparison of household-level and stratum-level regression approaches.

| Criterion | Household-level regression | Stratum-level regression |
| --- | --- | --- |
| Data richness | Even surveys for which the sampling frame is not a stratum (e.g. in Nigeria: see above) can be analysed through this approach, as long as the stratum to which survey observations belong is known.  Any surveys for which a dataset is not available or shared cannot be analysed. | Any surveys irrespective of dataset availability can be analysed, as the point estimate and standard error are known or can be worked out from other meta-data.  Surveys for which the sampling frame is not a stratum cannot be analysed through this approach; an alternative is to analyse all data at whatever level the surveys have been done, but this would generally result in far fewer data points and thus plausibly much less accurate models. |
| Robustness | Similar robustness properties to generalised linear models. | If OLS regression is used, key model assumptions may not be met. |
| Applicability | Generally applicable. | If data are sparse or highly non-normal, OLS may not be appropriate, requiring non-parametric solutions. |

### Sensitivity analysis: Under-estimation of mortality in surveys

The true death rate within the survey sample is equal to $y=\frac{d_{o}+d_{u}}{\Pi_{o}+\Pi_{u}}$ , where $d$ is deaths, $\Pi$ is person-time, $o$ means observed and $u$ unobserved (i.e. not reported). If we assume that, out of all deaths, $p_{u}$ are not reported, $d=d_{o}+d_{u}=d_{o}+dp_{u}$ , i.e. $d_{u}=d_{o}\frac{p_{u}}{1-p_{u}}$ . Accordingly,$\Pi_{u}=d_{u}T_{r}f_{r,u}$ , where $f_{r,u}$ is the fraction of the recall period that unobserved deaths spend on average within the surveyed household (we assume that $f_{r,u}=0.25$: if most unobserved deaths are among infants and neonates, many of these might have died shortly after birth and thus contributed little to overall person-time). For alternative values of $p_{u}$, we compute the total number of unobserved deaths $D_{u,s}$ for each survey $s$, and from this the mean $\bar{d}_{u,s,i}$ per household $i$ in the survey sample. We then simulate a large number of datasets by generating a random value of $d_{u,s,i}$ from a Poisson distribution with mean = $\bar{d}_{u,s,i}$ , and calculate the total $d_{s,i}$ and $\Pi_{s,i}$ for both children under 5y and all ages. We use these augmented datasets to carry out further estimation steps and observe median results of the simulations.

# R analysis scripts and input datasets

## Description of the scripts

See <https://github.com/francescochecchi/mortality_small_area_estimation>. Table S4 details key data management and analysis sub-steps implemented by each R script, and required input datasets (in practice these are read only once by the control script). We recommend only interacting with these R scripts through RStudio (<https://www.rstudio.com/>). The scripts should be run in sequence, as they build on each other. Users should not need to modify any of the code, with the exception of the following:

- Under ### Specifying parameters,
  - select the country of analysis (e.g. “nga” for Nigeria): make sure that all input datasets (except for the SMART survey datasets) are named “[xxx]_[dataset name].xlsx”, e.g. “nga_analysis_strata.xlsx”;
- As mentioned above, script 2 is not (yet) generic for any crisis setting; a country-specific script will thus need to be developed by the analyst, though methods and code sections could replicate those developed for Somalia or Nigeria (to be published separately).

With time, it is possible that some of the R packages used in the scripts will become obsolete, and as such errors may begin to appear in any of the scripts. This may require small code modifications.

## Input datasets

To enable analysis replication and show the required input dataset structure, sample datasets are provided on <https://github.com/francescochecchi/mortality_small_area_estimation>. These are sufficient to implement analysis for Somalia (2014-2018). Key input datasets include:

- <[xxx]_analysis_strata.xlsx>, which simply contains the list of administrative level 0 (if needed), 1 (e.g. state) and 2 (e.g. county) geographical strata; further levels may be added, but corresponding parameters should be added under the <general_parameters> worksheet of <[xxx]_analysis_parameters.xlsx>;
- <[xxx]_survey_metadata.xlsx>, which contains unique IDs and various meta-variables for each SMART survey included in the analysis;
- Individual raw datasets of SMART surveys, as exported using ENA software into .csv format (these should be named as per the unique survey ID variable in the <[xxx]_survey_metadata.xlsx> file, and stored in a sub-directory that must be called ‘~/survey_datasets’); no modifications are needed on the ENA-exported datasets, but we have encountered occasional errors (e.g. non-numeric entries or lone non-empty cells in unmarked columns): these will probably throw an error during execution of script 1, necessitating some investigation and manual data cleaning (script 1 will show the survey ID where the error occurs);
- <[xxx]_predictor_data.xlsx>: this file contains a master table of all predictor datasets, with characteristics of each and options for how to manage them (script 3), including reshaping and aggregation, imputation, smoothing, interpolation, creation of lags, etc.;
- <[xxx]_demog_data.xlsx>: while script 2 (population reconstruction) as mentioned below will need to be developed for each crisis, maintaining the same data input structure as shown in the sample dataset is recommended to avoid script execution problems; generally this will include a table of all population and displacement datasets used, a worksheet with specific demographic parameters (e.g. birth rate), and the datasets themselves;
- <[xxx]_analysis_parameters.xlsx>: this is the main file enabling the user to interact with the analysis, and consists of a <general_parameters> worksheet (needed for all scripts) where various parameters needed across the analysis are declared; a <predictor_parameters> worksheet (scripts 4-6) wherein all variables to be considered in model fitting should be listed, with options to force their inclusion or exclusion, retain specific lags, categorise them, consider them in interaction terms, etc.; a <counterfactual_parameters> worksheet (scripts 5-6) where best, worst and most likely case values for both predictors and population input datasets are declared, so as to create corresponding counterfactual scenarios; and a <sensitivity_parameters> worksheet (script 6) where the user can specify sensitivity ranges for specific datasets (identified by their R object name).

Generally, dictionaries within each file are not just informational, but also determine which specific variables within each worksheet are read by the scripts. As such, they should be modified with care, and any additional variable or parameter added to any worksheet should be reflected within the corresponding dictionary.

Table S4. Explanation of R analysis scripts.

| R script | Sub-steps implemented | Required data inputs† | Outputs | Notes |
| --- | --- | --- | --- | --- |
| mortality_sae_0_control_code.R | - Install R packages - Read input datasets - Read and declare parameters - Source other dependent scripts (below) | - <[xxx]_analysis_strata.xlsx> - <[xxx]_survey_metadata.xlsx> - Individual raw datasets of SMART surveys (see above) - <[xxx]_analysis_parameters.xlsx> - <[xxx]_predictor_data.xlsx> - <[xxx]_demog_data.xlsx> | None | Any dependent script can also run by executing the script itself or a portion of it. |
| mortality_sae_0_functions.R | - Declare bespoke functions used by different scripts |  | None |  |
| mortality_sae_1_manage_surveys.R | - Reanalyse each survey and estimate additional demographic indicators - Explore survey availability across crisis person-time - Prepare survey observations for further analysis steps | - <[xxx]_survey_metadata.xlsx> - Individual raw datasets of SMART surveys (see above) | - Re-analysed survey estimates - Graphs and table of descriptive survey patterns - Merged and reshaped survey observations | Would need to be modified if mortality sources other than SMART surveys are introduced. |
| mortality_sae_2_reconstruct_pop_[xxx].R | - Reconstruct population denominators for each stratum by combining census estimates, internal displacement and refugee data - Estimate the proportion of IDPs as well as in- and out-migration rates | - <[xxx]_analysis_strata.xlsx> - <[xxx]_analysis_parameters.xlsx> - <[xxx]_demog_data.xlsx> | - Reconstructed population (including under 5y) denominators and IDP figures / proportions - Graphs of trends in denominators | Crisis-specific at present. |
| mortality_sae_3_manage_predictors.R | - Merge predictors into one time series - Transform predictor values into rate - Visualise completeness and apply completeness cut-offs - Perform specified manual and automated imputations - Compute rolling means and lags - Smooth and/or interpolate - Prepare datasets for model fitting | - <[xxx]_analysis_strata.xlsx> - <[xxx]_analysis_parameters.xlsx> - <[xxx]_predictor_data.xlsx> | - Graphs of completeness and smoothed time series - Predictor values for each $kt$ - Average predictor values over the recall period of each survey (or survey stratum) | Automated imputation not fully tested. |
| mortality_sae_4_predictive_model.R | - Explore predictor distributions - Categorise predictors - Univariate analysis (categorical vs. continuous, best-fitting lag, screening out predictors with low association) - Brute force search across all candidate models - Select best fixed-effects model based on cross-validation - Explore interactions - Fit mixed model and select between fixed-effects only and mixed option - Calculate robust standard errors if fixed-effects model is selected - Compute and graph various metrics of model performance - Save model for subsequent steps | - <[xxx]_analysis_parameters.xlsx> - <[xxx]_predictor_data.xlsx> | - Graphs of predictor distributions. - Fit statistics for each model evaluated by brute force. - Cross-validation fit statistics for most promising models. - Goodness-of-fit graphs, performance metrics and saved fits for the best models. | Needs to be run twice, once for CDR and once for U5DR.  **Very computationally intensive** (at least 5-10 hours on a standard laptop). Execution time depends mostly on: (i) how many models are being evaluated by brute force; (ii) number of folds for cross-validation (recommend 10 folds maximum); (iii) whether mixed model is fit and selected as best model. |
| mortality_sae_5_estimate_mortality.R | - Create counterfactual datasets for each scenario - Implement excess death toll estimation for three counterfactual scenarios (best, worst, most likely) - Aggregate death toll estimates as desired and create graphs and tables | - <[xxx]_analysis_parameters.xlsx> - <[xxx]_demog_data.xlsx> | - Graphs, tables and datasets of actual, counterfactual and excess death tolls and rates, for all ages and under 5y, by stratum, time unit as well as higher aggregations and overall. | Section on creating counterfactual population denominators may need to be modified if the step 2 script cannot be harmonised with this script.  **Computationally intensive** if > 1000 bootstrap samples are drawn. |
| mortality_sae_6_sensitivity_analyses.R | - Sensitivity analysis of population and displacement data - Sensitivity analysis of under 5y mortality underreporting - Implemented by creating new input datasets that incorporate sensitivity assumptions, and re-running all analysis steps | - <[xxx]_analysis_parameters.xlsx> | - Graphs and datasets of death toll estimates for each set of sensitivity parameter values. - To reduce computational intensity, this script only computes point estimates for each set of sensitivity parameter values, and omits various optional sub-steps. | **Very computationally intensive** if > 10 sets of sensitivity values are investigated.  May require increasing memory allocation. Suggest attempting this step with a minimal range of sensitivity values first. |

† Not listed are various additional datasets and R objects that will have been generated by a previous script.

1. Prudhon C, de Radiguès X, Dale N, Checchi F. An algorithm to assess methodological quality of nutrition and mortality cross-sectional surveys: development and application to surveys conducted in Darfur, Sudan. Popul Health Metr. 2011;9:57. [↑](#footnote-ref-1)
